# A new challenge for data analytics: transposons

**DOI:** 10.1186/s13040-022-00294-x

**Published:** 2022-03-25

**Authors:** Ralf E. Wellinger, Jesús S. Aguilar–Ruiz

**Affiliations:** 1 Centro Andaluz de Biología Molecular y Medicina Regenerativa–CABIMER, Universidad de Sevilla – CSIC – Universidad Pablo de Olavide, Seville, 41092 Spain; 2 Department of Genetics, University of Seville, Seville, 41012 Spain; 3 School of Engineering, Pablo de Olavide University, Seville, 41013 Spain

The evolution of the Data Analytics field, both in its scientific dimension, i.e. Data Analytics Science (research and development of machine learning techniques), and in its engineering extension, i.e. Data Analytics Engineering (analysis, design, implementation and deployment of Data Analytics projects), has been uneven over the last four decades and, to a large extent, conditioned by the growth rate on the capacity to generate information. The global volume of information reaching the Internet has been steadily increasing, far exceeding linearity over the last decades (currently, global traffic is around 2EB/day). Apart from the recent technological development, which includes the widespread use of mobile devices, the reduction in cost of sensors, or the improved performance of IT infrastructures, all of which have led to massive data generation, we find a reliable picture of how the typology of data has evolved in the datasets that have been used by the scientific community as a basis for the comparative analysis of innovative algorithmic approaches in Data Analytics.

In particular, we refer to the size of a dataset as the number of values it contains, but it is usual, because of the underlying implications, to refer to the product of the number of examples (instances) and the number of attributes (variables). To cite well–known examples –with a few exceptions– we observe frequent datasets up to the 90s that do not exceed 10,000 examples and 25 attributes (Iris [1], with 150×4; Hepatitis [2], with 155×19; Lenses [3]], with 24×4; or Mushrooms [4], with 8,124×22). However, between 1990 and 1999 there was an increase in the number of examples, exceeding tens of thousands (Letter recognition [5], with 20,000×16; Adult [6], with 48,842×14), although the number of attributes remained similar. In 2000 the challenge of analyzing a dataset with 4 million examples and 42 attributes was launched [7] (this led to coining of the term Large Dataset [8], which the same authors limited to 1000MB).

With the arrival of the new millennium, the number of instances began to explode, to the extent that some cases became unmanageable due to the speed of arrival, consolidating a new discipline known as data streams [9] (continuous data flows, which are distributed over time with unequal intensity –number of instances at a given time– and undefined periods or time stamps –variable arrival frequency–) that remains strong today. Several works appeared then, among them [10–15] that tried to define techniques (classification and clustering) capable of providing good results for dynamic volumes of data, as opposed to techniques designed for static analytics (where all data is available before the execution of the model). Currently, advances in data streams –which require greater scientific depth in the handling of incrementality– have not been as spectacular as previously foreseen, possibly due to the irruption of Big Data, which prioritized the interest in infrastructures and parallelism to the detriment of algorithms. However, in one way or another, hundreds of thousands or millions of data are now no obstacle, even for techniques of greater algorithmic complexity, such as those covered by Deep Learning, which show some ability with invariant operations, but are generically placed in NP–hard or coNP–complete categories [16]. In any case, it is not common for biological or clinical data to be generated at high speed, following the typology of data streams, with the exception of those coming from health sensors (wearable, implanted or digestible), helping bring healthcare anywhere and anytime.

In short, there is an inflection in the research trend, redirecting scientific attention towards the quasi–optimal use of computing resources, which justified, to a certain extent, the use of techniques with higher than quadratic computational cost (e.g., those based on Deep Learning). However, this trend is theoretically limited by Amdahl’s Law [17] and, more precisely, by Gustafson’s Law [18], so that when dealing with huge amounts of data, the use of resources with multiple cores (CPUs, GPUs or TPUs: Central, Graphics or Tensor Processing Units, respectively) or software technologies (CUDA, OpenCL, Spark or PlaidML) can reduce processing times, but in many cases, only anecdotally (dividing the sequential run time by a constant).

The genomics boom, and in particular the data–generating capacity of microarray–based techniques, fostered the appearance of datasets that, unlike the previous ones, contained very few examples and thousands of attributes. Typically, the examples were associated with samples or patients, and the attributes with gene expression levels. The first popular datasets emerged in the late 90s (yeast [19], with 17 examples and 2,884 genes; leukaemia [20], with 38 examples and 6,817 genes; lymphoma [21], with 96 examples and 4,026 genes; among others). The evolution and affordability of sequencing techniques led to a massive generation of data, as well as to the overflow of analysis capacity and, therefore, established a bottleneck in biological research [22] that demanded a greater involvement of Data Analytics. As an example, if the complete genome of a species cost about $100M in 2000, since mid–2015 it has been around $1,000 and, very soon, a human genome will be worth less than $100. Logically, more relevant than the volume itself is the proliferation of studies aligned with the so–called Precision Medicine or Personalized Healthcare, and the notable impact it will have on society in the years to come.

The number of variables involved exceeded tens of thousands, without increasing the number of examples, accentuating the well–known problem of “the curse of dimensionality”. This is a major obstacle with this typology of data, as a minimal representation of values for a variable requires at least two extremes close to the limits of the variable’s range, and some intermediate value. For each variable, three values translates into a Cartesian product of the dimensions involved, i.e. 3^*n*^ values to have that minimum good representation of the n–dimensional search space. In short, if n=100, we would need more than 10^47^ values and, if the number of variables reaches thousands or tens of thousands, the difficulty is as great as unimaginable. The “curse” is more than justified, and causes severe consequences in predictive (e.g. classification or regression) or descriptive (e.g. clustering or biclustering) models. Adversely and further complicating matters, an extraordinary actor has appeared in the analytical landscape: the transposon.

For decades, about 98 per cent of our DNA was termed “junk” based on the fact that it did not code for proteins. Some content of this “junk DNA”, namely transposons, is suitable to illustrate the complexity of data analysis linked to an infinite variables. Transposons (also called the “jumping genes” or transposable elements: TE) were discovered by Barbara McClintock in 1948 in maize plants, and after many years of skepticism that mobile DNA elements are important for genetic regulation of the eukaryotic genome, her findings were awarded with an unshared Nobel Prize in Medicine in 1983. In short, a transposon is a DNA fragment that can duplicate and change its position in the genome, and thereby modify genome organization and functionality. About half of the human genome is composed of transposons [23] explaining their important role for providing human genetic diversity. The impact of transposons is fascinating: they regulate gene transcription, can activate the immune system (see autoimmune diseases), connect regulatory networks, modulate protein structure (see neurodegenerative diseases), are associated with hereditary cancer or contribute to the emergence of new genes, among others [24]. On the other hand, transposons are potent tools for gene therapy [25, 26] or the discovery of drug targets, for example by saturated transposon analysis in budding yeast [27]. Simple, eukaryotic model systems such as yeast are still advantageous for massive sequencing approaches and data analysis. In contrast to yeast, the complexity of the human genome hampers application of transposons in Data Analytics and Personalized Medicine [28]. Given that transposons are the most abundant elements in the genome, transponsons provide an excessive amount of variables (polymorphisms) linked to cell type, chromatin organization, gene regulation or gene functionality, posing a serious threat to data analysis. Transposons are polymorphic elements and individuals may either be homo– or heterozygotic for a given polymorphism. To provide a glimpse on the complexity of transposon analysis, we would like to mention a recent study of long interspersed element–1 (LINE-1) transposon profiling in human somatic cells to reveal LINE-1 insertion sites linked to ovarian cancer development [29]. In order to carry out this study, a machine–learning–based computational pipeline was developed to identify insertion sites derived from next–generation sequencing data. Another example for the complexity of data analysis is the study of transposable element expression dynamics and heterogeneity at the single–cell level during development of mouse embryonic stem cells (mESCs) by RNA sequencing [30]. In order to integrate the data, the authors had to develop a data processing pipeline based on an algorithm that quantifies transposon expression in single–cell sequence data.

It is not the purpose of this paper to dwell on biological or clinical aspects, but rather on the implications of the transposon for the future of Data Analytics and Personalized Medicine, as information is also recently being generated for transposons. Thus, if the dimensionality was in the order of tens of thousands, it has been extended to several millions of variables due to transposons. At scientific level, the difficulties involved in analyzing datasets with a few thousand examples and millions of variables are of high magnitude. In fact, most algorithms would not even be useful because they are inefficient due to their algorithmic complexity or because they include elements that force the use of all variables –e.g. metrics between instances–, i.e. they would be computationally intractable. Not even Fugaku, the world’s most powerful supercomputer –as of December 2021–, with 442 PFlops, and more than 7.5 million cores, would be able to execute a exhaustive search in the hyperdimensional space defined by millions of variables. Therefore, the importance that progress in descriptive or predictive techniques with good performance in hyperdimensional contexts would reveal is very significant, as hard technologies facilitate very much, but they will not solve.

A hypothetical standard scenario of 1,000 samples and 4 million variables means analyzing 4 billion values in the best case (computational time complexity is *O*(*nm*), where *n* is the number of samples and *m* is the number of variables). However, this scenario has not only a sight but different perspectives. Let *t* be a test example to be classified by a predictive model *M* learned from the dataset *D* with *n*×*m* values. The computational cost of training the model *M* from *D* in order to predict the unknown class of *t* is highly depending on the machine learning technique chosen. For example, the nearest neighbor (NN) technique would require to calculate a distance metric from *t* to any *e*∈*D*, with time complexity in *O*(*nm*). Similarly, the Naïve–Bayes (NB) technique would have a similar cost, although repeating the process for each class label of the target variable, with time complexity in *O*(*knm*), where *k* is the number of class labels. Both techniques are very sensitive to the curse of dimensionality, since the relevance of a given variable would be completely diluted in the calculation of the distance (NN) or of the probabilities (NB). In contrast, decision tree–based techniques build predictive models with slightly higher complexity *O*(*nm* log2*n*), but the prediction of *t* only depends on a subset of variables (not all), which alleviates the curse of dimensionality. More sophisticated approaches based on Deep Learning, would need a huge input layer (*m* neurons). Therefore, the number of trainable parameters of the neural architecture would reach unmanageable numbers (only one fully–connected hidden layer with *n* neurons and an output layer with *k* neurons –for softmax activation– would lead to *mn*+*nk* parameters), to which must be added the neural learning process itself over a number *p* of epochs dealing with *n* samples each (or for mini–batch *n*^′^<*n*). Complex deep learning architectures, with several hidden layers, could easily reach trillions of parameters in this highly dimensional context.

This genomic landscape of each person is different and determines the response to external stimuli, medication or human disease prediction. To advance the application of omics datasets in Personalized Medicine, machine learning approaches will be required to channel data that contain millions of variables and unpredictable outcomes into a feasible risk assessment that allows an optimal treatment of individual patients. Transposon derived data undoubtedly pose a huge scientific challenge for Data Analytics, but in combination with other omics data, in the near future they will help to drive significant, scientific advances on the understanding and treatment of human disease.

## Data Availability

Not applicable.
